# Malnutrition Risk Among Hospitalized Patients With Type 2 Diabetes Mellitus and Its Association With Hospital Length

**DOI:** 10.1002/puh2.70011

**Published:** 2024-11-27

**Authors:** Philemon Kwizera, Reverien Niyomwungeri, Omar Gatera, Harriet Gyamfuah Adu‐Amoah, Jeannine Ahishakiye

**Affiliations:** ^1^ Human Nutrition and Dietetics Department, College of Medicine and Health Sciences (CMHS) University of Rwanda Kigali Rwanda; ^2^ Internal Medicine Department University Teaching Hospital of Kigali Kigali Rwanda; ^3^ African Center of Excellence in Internet of Things (ACEIoT) College of Science and Technology University of Rwanda Kigali Rwanda

**Keywords:** diabetes mellitus Type 2, hospital malnutrition, length of stay, nutrition risk index

## Abstract

**Background:**

Estimating malnutrition risk among hospitalized patients is challenging, yet critical due to its association with adverse outcomes such as prolonged hospital stays, increased mortality, impaired wound healing, depression, and increased hospital costs. This research study aims to evaluate the risk of malnutrition among Type 2 diabetic hospitalized patients and its impact on the patient's length of stay in two tertiary hospitals based in Kigali.

**Method:**

In this retrospective cross‐sectional study, 300 adult hospitalized Type 2 diabetic patients from Kigali's tertiary hospitals were enrolled between January 2021 and October 2022. Data collected include demographics, anthropometrics, serum albumin, and length of hospital stay. The nutrition risk index was used to determine malnutrition risk.

**Results:**

Overall, 55.3% of hospitalized Type 2 diabetes patients was found to be at risk of malnutrition on the basis of the nutrition risk index. Gender distribution showed no significant difference (*p* = 0.724), with 56.6% females and 54.5% males at risk. Significant associations were found with age (*r* = 0.018, *p* = 0.017), hypertension as comorbidity (*r* = ‐0.169, *p* = 0.004), hospital stay duration (*r* = 0.139, *p* = 0.002), and blood glucose levels (*r* = −0.087, *p* = 0.001).

**Conclusion:**

A study finds high malnutrition risk in hospitalized Type 2 diabetes patients, linked to longer stays and poor outcomes. Early malnutrition screening, proper nutrition support, and a multidisciplinary care team are crucial for improved clinical care and cost‐effectiveness.

## Graphical abstract

This study assessed malnutrition risk among hospitalized Type 2 diabetic patients in Kigali's tertiary hospitals. Among 300 patients, 55.3% was at malnutrition risk. Gender distribution did not differ significantly, but age, hypertension, longer hospital stays, and higher blood glucose levels were associated with malnutrition risk.

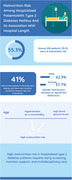

## 1 Introduction

Malnutrition is a significant issue within hospitals, impacting approximately 20%–50% of patients globally [1], and it is associated with numerous adverse clinical outcomes such as high morbidity and mortality rates, extended hospital stays, susceptibility to infections, loss of muscle mass, and inadequate wound healing [2]. Malnutrition is defined as insufficient, excessive, or disproportionate consumption of energy and/or essential nutrients by an individual [3].

Type 2 diabetes, also known as diabetes mellitus (DM), is a chronic metabolic disorder characterized by the body's reduced ability to utilize the hormone insulin efficiently and/or inadequate insulin production [4]. This condition represents a remarkable worldwide public health concern among adults 20–79 years, with an estimated prevalence of 10.5% (equivalent to 537 million individuals) in 2021 [5]. Projections indicate that the prevalence is expected to increase to 11.3% (approximately 642.7 million) by 2030 and further to 12.2% (roughly 783.2 million) by 2045 [6].

Diabetes is acknowledged as a significant cause of early death and disability. It is one of the four priority noncommunicable diseases (NCDs) highlighted by global leaders in the 2011 Political Declaration on the Prevention and Control of NCDs [7], and the declaration emphasizes that the incidence and impact of diabetes can be significantly prevented or reduced through the implementation of evidence‐based, affordable, and cost‐effective strategies. These strategies should be applied on a large scale and involve multiple sectors working together [8]. Hence, we aim to call on researchers and other stakeholders to actively contribute to the development and implementation of these strategies with a particular emphasis on hospitalized patients who often face challenges in improving their nutritional status and are at risk of being overlooked in current interventions.

According to the second Rwanda NCDs risk factors study, the prevalence of diabetes has remained at 3% over the last 9 years. Especially, Kigali City exhibits the highest diabetes prevalence, standing at over four times higher than the national average of 9.8% [9]. Ineffective nutritional screening on admission and insufficient food intake among patients who are hospitalized are frequently linked to negative health outcomes [10].

Several factors contribute to insufficient food consumption among hospitalized patients, including the absence of feeding assistance, challenges in delivering regular nutritious meals, and meal omissions due to clinical examinations and procedures [10]. The nutritional status of patients, whether normally nourished or undernourished, can often worsen during hospital admission. This decline can be attributed to various factors, including the physiological and metabolic effects of the illness itself, as well as a decrease in food intake relative to the body's requirements [11].

In Southern Ethiopia, a cross‐sectional study carried out in East Africa unveiled an overall hospital prevalence of malnutrition at 25.2%. Among the participants, 49 individuals were classified as mildly malnourished, 19 as moderately malnourished, and 9 as severely malnourished [12].

As far as we know, there has not been a study in Rwanda examining the occurrence of malnutrition among diabetic patients admitted to hospitals. Therefore, this study aimed to determine the risk of malnutrition among hospitalized patients diagnosed with Type 2 DM and its association with hospital length in tertiary hospitals based in Kigali.

## 2 Materials and Methods

### 2.1 Study Design and Setting

All the patients with Type 2 DM who were hospitalized in the internal medicine department in two Referral Hospitals based in Kigali (University Teaching Hospital of Kigali and King Faysal Hospital) for poor glycemic control (high glucose levels) between January 2021 and October 2022 were recruited.

### 2.2 Study Population and Sample

Secondary data were collected from patients aged above 18 who were hospitalized due to diabetic complications. Patients without measurement of albumin level were excluded from this analysis. We also excluded patients with missing data on follow‐up. The sample size of 300 hospitalized patients was calculated using the STEPS sample size calculator of WHO and on the number of yearly hospital admissions with a 95% confidence interval with a margin of error of 0.05 and a 100% expected response rate [13].

### 2.3 Data Collection Methods

Patient demographics, anthropometric and clinical characteristics such as age, gender, admission diagnosis, date of hospital admission and discharge, weight (present and usual weight), height, glucose levels, and serum albumin were retrieved from the patients’ hospital records. Body mass index (BMI) (weight kg/height m^2^) was calculated and categorized as follows: BMI <18.5 indicates underweight, BMI 18.5–24.9 signifies normal weight, BMI ≥25.0 suggests overweight, and BMI ≥30.0 denotes obesity [14]. The length of hospital stay was calculated from the date of admission to the date of discharge, and it was categorized according to the cutoff point established in the literature as a very long hospital length (≥16 days) [15].

### 2.4 Assessment of Nutrition Risk Index

The calculation for the nutrition risk index was performed using the equation: NRI = (1.519 × serum albumin in grams per liter) + (41.7 × present weight divided by usual weight). Patients with an NRI score higher than 100 were categorized as being in the no‐risk group. Scores ranging from 97.5 to 100 were considered mild risk, whereas scores between 83.5 and 97.5 were classified as moderate risk, and scores below 83.5 were categorized as severe risk groups. However, the mild risk, moderate risk groups, and severe risk groups were combined [16].

### 2.5 Data Analysis

Data obtained were entered and analyzed using the Statistical Package of Social Sciences (SPSS) software version 27. Descriptive data were presented as tables. The categorical variables studied were expressed as proportions and percentages. Associations between categorical variables were analyzed using Chi‐square. Spearman's correlation was used to test the association between variables. A *p* value less than or equal to 0.05 was considered statistically significant, and the retrospective design posed challenges like missing data and biases. We mitigated these using imputation methods and sensitivity analyses to ensure result robustness.

### 2.6 Ethical Considerations

The study protocol was approved by

· IRB CMHS: November 14, 2022 (Approval notice: CMHS/IRB/500/2022)
· KFH IRB: December 30, 2022 (Approval Ref KFH/2022/ 035/IRB)
· CHUK ethics committee: March 14, 2023 (Approval Ref EC/CHUK/046/2023)

The data collectors ensured participants' confidentiality and privacy by not keeping personal identification information; instead, they used a code in place of the name of each participant.

## 3 Results

### 3.1 Background and Clinical Characteristics of the Enrolled Patients

Table 1 presents information on 300 patients hospitalized with Type 2 DM between January 2021 and October 2022. The patients' ages ranged from 20 to 90 years, with a mean age of 54.27 years and a standard deviation of 16.3 years. The majority fell above the 61 age range of 123 patients (41%), and 87 patients (29%) fell into the age category of less than 45 years, whereas 90 patients (30%) belong to the age range of 46–60 years. In terms of gender distribution, 113 (37.7%) were female, whereas 187 (62.3%) were male. Weight data revealed a present weight range of 38.0–113.0 kg, with a mean of 74.7 kg, and a usual weight range of 35.0–109.0 kg, with a mean of 74.2 kg. The patients' heights ranged from 1.5 to 1.98 m, with a mean of 1.67 m.

**TABLE 1:** Background and clinical characteristics of the enrolled chronic patients in the internal medicine department in two Referral Hospitals based in Kigali, 2022.

Variables	*N* = 300 (*n* [%])
**Age (years)**	
Range	20.0–90.0
Mean ± SD	54.27 ± 16.3
<45	87 (29)
46–60	90 (30)
>61	123 (41)
**Sex**	
Female	113 (37.7)
Male	187 (62.3)
**Present weight (kg)**	
Range	38.0–113.0
Mean ± SD	74.7 ± 15.7
**Usual weight (kg)**	
Range	35.0–109.0
Mean ± SD	74.2 ± 15.4
**Height (m)**	
Range	1.5–1.98
Mean ± SD	1.67 ± 0.08
**BMI (kg/m^2^)**	
Underweight	28 (9.3)
Normal weight	102 (34)
Overweight	78 (26)
Obesity	92 (30.7)
**Blood sugar level (mg/**dL)	
Range	33.5–898.0
Mean ± SD	215.9 ± 137.9
**Albumin (g/**dL)	
Range	18.0–77.3
Mean ± SD	37.3 ± 6.7
**Hypertension**	
Yes	183 (61)
No	117 (39)
**Hospital stays (days)**	
Range	0.0–182.0
Mean ± SD	15 ± 23
Nonprolonged <16 days	152 (50.7)
Prolonged HS ≥16 days	148 (49.3)
	

BMI analysis classified 9.3% of patients as underweight, the majority were 34% as normal weight, 26% as overweight, and 30.7% as obesity. Blood sugar levels varied widely, ranging from 33.5 to 898.0 mg/dL, with a mean of 215.9 mg/dL. The albumin level ranged from 18.0 to 77.3 g/dL, with a mean of 37.3 g/dL. Hypertension was prevalent among 61% of patients, whereas 39% did not have hypertension. Hospital stays ranged from 0 to 182 days, with a mean stay of 21.9 days and a standard deviation of 26.0 days, furthermore, regarding the duration of hospital stays for the patients with Type 2 DM. Among the total, 152 patients (50.7%) had nonprolonged hospital stays, defined as less than 16 days, whereas 148 patients (49.3%) experienced prolonged hospital stays of 16 days or more.

### 3.2 Prevalence of Malnutrition Risk

Table 2 presents data on the nutrition risk index of 300 patients who were hospitalized with Type 2 DM.

**TABLE 2:** Nutrition risk index in the internal medicine department in two Referral Hospitals based in Kigali, 2022.

Variables	Frequency	Percentage
At risk of malnutrition	166	55.3
No risk of malnutrition	134	44.7
Total	300	100

The results show that we have a prevalence of malnutrition of 55.3%, whereas 44.7% had normal nutritional status.

### 3.3 Malnutrition Risk and Different Backgrounds and Clinical Characteristics

Table 3 indicates associations between malnutrition risk and various variables among hospitalized patients. Gender distribution showed no significant difference between those at high risk of malnutrition and those not at risk (*p* = 0.724). Observed numbers revealed 64 females (56.6%) and 102 males (54.5%) at risk, compared to 49 females (43.4%) and 85 males (45.5%) not at risk.

**TABLE 3:** Distribution of the enrolled patients by malnutrition risk and different backgrounds and clinical characteristics in the internal medicine department in two Referral Hospitals based in Kigali, 2022.

	At risk of malnutrition (n [%])		*p* value
Variables	Yes (*N* = 166)	No (*N* = 134)	
Sex			0.724
Female	64 (56.6)	49 (43.4)	
Male	102 (54.5)	85 (45.5)	
Age			
<45	48 (55.2)	39 (44.8)	0.017
46–60	48 (53.3)	42 (46.7)	
>61	70 (56.9)	53 (43.1)	
**Hypertension**			
No	77 (65.8)	40 (34.2)	0.004
Yes	89 (48.6)	94 (51.4)	
Hospital stay			
Nonprolonged <16 days	59 (38.8)	93 (61.2)	0.002
Prolonged ≥16 days	107 (72.3)	41 (25.7)	
BMI			
Underweight	20 (71.4)	8 (28.6)	0.311
Normal	54 (52.9)	48 (47.1)	
Overweight	44 (56.4)	34 (43.6)	
Obesity	48 (52.2)	44 (47.8)	
Blood sugar level (mg/dL)	215.2 ± 155.6	206.7 ± 112.7	0.001

In contrast, significant associations with positive correlation as indicated in Table 4 were found between age and malnutrition risk: <45 years (55.2% at risk), 46–60 years (53.3% at risk), and >61 years (56.9% at risk). Individuals aged <45 years exhibited a statistically significant difference in malnutrition risk compared to older age groups (*r* = 0.018, *p* = 0.017). Hypertension also showed significance with negative correlation (*r* = −0.169, *p* = 0.004), with observed numbers indicating 77 (65.8%) and 89 (48.6%) patients at risk with and without hypertension, respectively.

**TABLE 4:** Correlations between malnutrition risk as revealed by different background and clinical characteristics in the internal medicine department in two Referral Hospitals based in Kigali, 2022.

Variables	Malnutrition risk
Age	
*r*	0.018
*p*	0.017
Hypertension	
*r*	−0.169
*p*	0.004
Hospital stay	
*r*	0.139
*p*	0.002
Blood sugar level (mg/dL)	
*r*	−0.087
*p*	0.001

**FIGURE 1:**
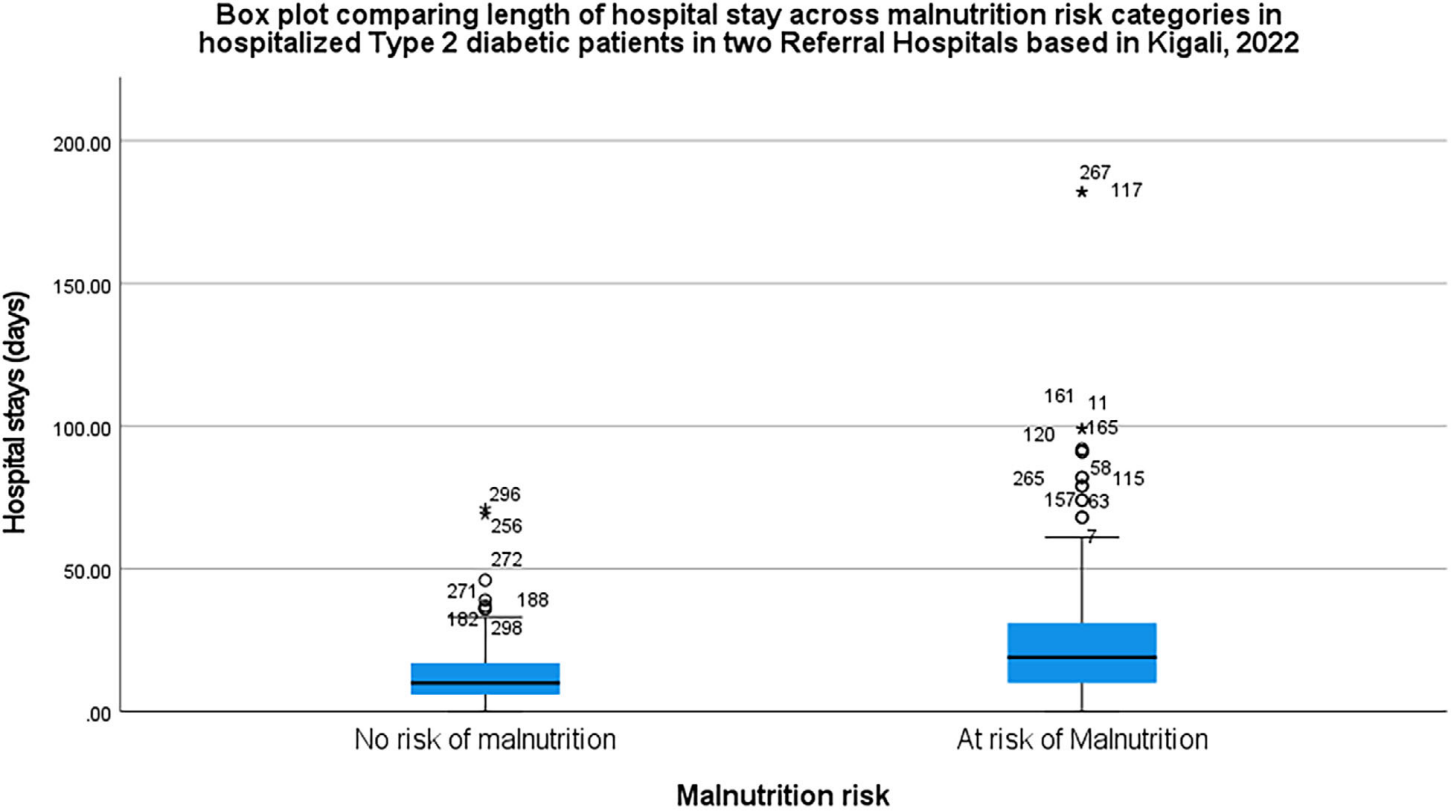
Box plot comparing length of hospital stay across malnutrition risk categories in hospitalized Type 2 diabetic patients in two Referral Hospitals based in Kigali, 2022.

Hospital stay duration also showed positive correlation significance (*r* = 0.139, *p* = 0.002), with high numbers of patients who had prolonged stay (≥16 days) in hospital with 107 (72.3%) compared to 59 (38.8%) patients who did not have prolonged stay (<16 days), and both are at risk of experiencing malnutrition. In addition, Figure 1 shows that hospitalized Type 2 diabetic patients at risk of malnutrition have a higher median length of stay and greater variability in hospital stay duration. Regarding BMI categories, no significant differences were observed (*p* = 0.311), with observed numbers indicating 20 (71.4%), 54 (52.9%), 44 (56.4%), and 48 (52.2%) patients at risk of falling into the respective underweight, normal weight, overweight, and obesity categories. Blood sugar levels, however, exhibited a negatively significant correlation difference (*r* = ‐0.087, *p* = 0.001), with mean levels of 215.2 and 206.7 mg/dL for those at high risk and those not at risk, respectively.

## 4 Discussion

The main objective of the study was to assess the malnutrition risk prevalence among hospitalized patients with Type 2 DM, and we employed the documentation approach in a cross‐sectional study that included a sample size of 300, representing the population. The study area was Referral Hospitals based in Kigali. The current study revealed that within the Internal Medicine Department, 55.3% of enrolled patients was at risk of malnutrition according to the nutrition risk index criteria. Factors that were associated with malnutrition risk among the patients included age, extended hospital stays, and the presence of comorbidities (hypertension) and elevated blood glucose levels.

A total of 113 (37.7%) were female, whereas 187 (62.3%) were male. Most of the patients in the study were elderly (41%), and there was a positive correlation between age and malnutrition. The elderly population is vulnerable to malnutrition due to a variety of factors, such as physical weakness due to muscle loss, taking multiple medications, the overall decline in health, cognitive decline, decreased appetite, reliance on others for food intake, difficulty in swallowing and chewing, confusion, and constipation [17]. A study done in Nigeria reported that malnutrition was 7.3% significantly higher among the elderly with T2DM [18].

Patients identified as malnourished according to the nutrition risk index criteria experienced a significantly longer hospital length of 16 days and were older. Both hospital length and advanced age serve as proxies for a patient's clinical outcomes and economic hardship [19]. Patients with malnutrition tend to have a longer length of stay in the hospital compared to those who are well‐nourished [17] as indicated in this study. Patients who prolonged (≥16 days) hospital stay had a high risk of malnutrition of 107 (72.3%) with a significance of (*p* = 0.002) and a positive Spearman correlation of 0.139. This robust correlation links malnutrition with unforeseen complications and deteriorating clinical conditions, underscoring the urgency of early malnutrition screening and detection during hospitalization.

This is because malnutrition can weaken the immune system, increase the risk of infection, and delay wound healing, which can all contribute to a longer hospital stay [20]. Prolonged hospitalization can lead to decreased appetite, impaired nutrient absorption, and increased nutrient losses, which can result in malnutrition (39). Conversely, low BMI was the least prevalent criterion in our sample population. More than half of the malnourished patients (54, 52.9%) exhibited a normal BMI, underscoring the imperative of not exclusively depending on BMI for nutrition assessment, a challenge frequently confronted by clinicians [19].

In this study, a significant association was observed between poor glucose control and risk of malnutrition (*p* value = 0.001, the Spearman correlation = −0.087). Patients with DM who exhibit poor compliance with their glucose‐lowering medications are at increased risk of poor glycemic control and the development of chronic DM complications, such as autonomic neuropathy and diabetic kidney disease [21]. These complications may predispose them to malnutrition. This finding aligns with a report by Woo et al., which indicated that elderly individuals with DM experiencing poor glycemic control are at a heightened risk of malnutrition [22]. Future studies should aim at developing interventions to reduce malnutrition in hospitalized diabetic patients, alongside improved nutrition screening and multidisciplinary care approaches. There is also a need for studies focused on a prospective design to reduce recall bias and strengthen the validity of the results.

The limitation of the study is the relatively small sample size; although the sample size was sufficient to detect malnutrition risk, it was too small for subgroup analyses, preventing us from identifying potential associations. However, the strength of this study lies in the fact that this is the first study to the best of our knowledge that assessed malnutrition risk in hospitalized patients with Type 2 diabetes in tertiary hospitals based in Kigali.

## 5 Conclusion

Our study reveals a high prevalence of malnutrition risk among hospitalized patients with Type 2 diabetes on admission which is directly associated with prolonged length of stay and worsening clinical outcomes. Recognizing the critical role of early malnutrition screening and assessment of patients with the right nutrition support during admission is a very critical component of clinical care. On top of that, a multidisciplinary care team, including registered dieticians and nutritionists’ approach to care, is highly recommended and remains a practical cost‐effective approach in patient care.

## Author Contributions

P.K. designed the study protocol, coordinated data collection, analyzed the data, and wrote the manuscript. J.A. and O.G. contributed to the design of the study protocol, guided the analysis and the writing of the manuscript, reviewed the manuscript, and approved it for submission. R.N. coordinated data collection and reviewed the manuscript. H.G.A.‐A. contributed to the review of the manuscript. All authors read and approved the final manuscript.

## Conflicts of Interest

The authors declare no conflicts of interest.

## Data Availability

The authors can provide the data of this research on reasonable request.
